# Corrosion Behavior of CMT Cladding Layer of AZ91 Magnesium Alloy Subjected to Friction Stir Processing

**DOI:** 10.3390/ma17122875

**Published:** 2024-06-12

**Authors:** Yang Chen, Junqi Shen, Shengsun Hu, Yahui Zhen, Huichao Zhao

**Affiliations:** 1 Tianjin Key Laboratory of Advanced Joining Technology, Tianjin University, Tianjin 300354, China; chenyang236@tju.edu.cn (Y.C.); huss@tju.edu.cn (S.H.); 15102220135@163.com (Y.Z.); zhao4672262@gmail.com (H.Z.); 2 School of Materials Science and Engineering, Tianjin University, Tianjin 300354, China

**Keywords:** friction stir processing, AZ91 magnesium, CMT cladding, corrosion behavior, grain refinement, β-Mg_17_Al_12_ second phase

## Abstract

Friction stir processing (FSP) was performed on an AZ91 magnesium alloy cladding layer fabricated by a cold metal transfer (CMT) technique. Electrochemical properties and immersion corrosion behavior of the cladding layer before and after FSP in 3.5 wt.% NaCl solution were investigated. After applying the FSP, the corrosion potential and corrosion current density of the cladding layer increased from −1.455 V to −1.397 V and decreased from 4.135 μA/cm^2^ to 1.275 μA/cm^2^, respectively. The results of OM and SEM displayed the refinement of grains and the dispersion of β-Mg_17_Al_12_ second phase in the friction stir processed (FSPed) cladding layer and more severe corrosion of the unprocessed sample. The corrosion rate of the FSPed cladding layer was lower, and a more compact corrosion product film was formed on the surface of the FSPed cladding layer. EDS results and XRD patterns showed that the corrosion products was mainly composed of Mg(OH)_2_. The increase in Al content in the α-Mg matrix, grain refinement, and fragmentation and dispersion of the β-Mg_17_Al_12_ second phase induced by FSP were the main factors that led to the improvement in corrosion resistance of the cladding layer of the AZ91 magnesium alloy fabricated by CMT.

## 1. Introduction

Magnesium alloys, readily available and the lightest metal structural materials for engineering applications, have the characteristics of superior specific strength, specific stiffness, aging resistance, thermal conductivity, and recyclability. Thus, magnesium alloys have shown great application value and potential in the fields of 3C (computers, communications, and consumer electronics), automobile, aerospace, and biomedical and energy sectors, etc. [1,2]. However, magnesium is susceptible to corrosion due to its active chemical characteristics, especially serious pitting corrosion in environments with the presence of Cl^−^ [3,4]. It has become a research hotspot to develop technical solutions to repair the damage to components made of magnesium alloy induced by corrosion and even enhance their properties. The cold metal transfer (CMT) technique, processing the characteristics of low heat output and a stable welding process without spatters during a droplet short circuit transfer process, is ideal for repairing damaged structures made of magnesium alloy. Friction stir processing (FSP) is a new type of solid-state processing technology derived from friction stir welding (FSW), and can be used to obtain fine-grained microstructures that are not easily obtained by other fusion welding methods and provide improved performance over traditional processing technologies [5,6]. FSP can lead to the breakup of the coarse second phases and refine the microstructure of alloys through the plastic deformation, mixing, crushing and dynamic recrystallization in the processing area induced by the high-speed rotation and movement of the stirring head [7,8].

The FSP technique has already been applied to many types of Mg alloys, such as WE43 [9], AZ91 [10], AZ31B [11], AZ80 [12], and AE42 [13], as well as a magnesium matrix composite [14], in order to improve their microstructure, mechanical properties, and corrosion resistance. Wu et al. [15] found that the friction stir processed (FSPed) sample of a WE43 Mg alloy had higher corrosion resistance than that of the base metal (i.e., unprocessed sample) due to its finer grains. Kumar et al. [16] investigated the microstructural and corrosion properties of pure magnesium and Mg–hopeite composites fabricated by FSP (i.e., one-pass, two-pass, and three-pass FSP), and the results showed that the three-pass FSP Mg–hopeite composite with the finest grain size had the lowest corrosion current density (1.1204 × 10^−5^ A/cm^2^) and the highest corrosion potential (−1.5129 V), demonstrating that it had the best corrosion resistance. As reported by Patel et al. [11], a stationary shoulder friction stir processed (SSFSPed) AZ31B magnesium alloy developed a fine uniform equiaxed grain structure across the thickness of the stir zone (SZ), which reduced the galvanic corrosion tendency, thus showing a relatively uniform corrosion performance with mud cracking and intergranular corrosion patterns different from the severe pitting corrosion of the base metal (BM). A study by Baradarani et al. [17] showed that ultrasonic-assisted FSP (UaFSP) can increase the open circuit potential of an AZ91 magnesium alloy, and the current corrosion density of the UaFSPed sample (2.09 μA/cm^2^) was much lower than that of the BM (6.82 μA/cm^2^), indicating that UaFSP could significantly improve the corrosion resistance of AZ91 Mg alloy. According to Liu et al. [18], localized corrosion predominated in the corrosion morphology of AZ91 magnesium alloy specimens before/after FSP, and FSP substantially decreased the degree of corrosion and reduced the corrosion rate of AZ91 Mg allay in 3.5 wt.% NaCl solution. Based on corrosion immersion tests and electrochemical corrosion tests performed by Arora et al. [19], the corrosion rate of an AE42 magnesium alloy was significantly decreased after FSP, which was induced by grain refinement and fine second-phase precipitation.

As aforementioned, the majority of research has focused on the effects of FSP on the microstructure and corrosion properties of the as-cast magnesium alloys. In this study, the electrochemical performance and immersion corrosion behavior of an AZ91 Mg alloy CMT cladding layer before and after FSP were investigated, and the corresponding corrosion mechanism was also analyzed systematically.

## 2. Experimental Procedure

The as-cast AZ91 magnesium alloy plate with a size of 250 mm × 150 mm × 6 mm and AZ91 magnesium alloy wire with a diameter of 1.2 mm were selected as the substrate and deposited material for the CMT arc cladding process, respectively. The chemical compositions of the substrate and deposited wire are shown in Table 1.

The arc cladding experiment of the AZ91 magnesium alloy was conducted by using a CMT arc cladding system, consisting of an industrial six-axis robot (MOTOMAN HP6, YASKAWA Electric Corporation, Fukuoka, Japan) and a digital welding machine (Fronius CMT Advanced 4000R, Fronius Intelligent Equipment (China) Co., Ltd., Shanghai, China), and the magnesium alloy welding procedure (DC-CMT-C1068, Fronius Intelligent Equipment (China) Co., Ltd., Shanghai, China) was adopted. During the CMT cladding process, pure argon was used as the shielding gas with a flow rate of 15 L/min, and the wire feed speed and welding speed were 12 m/min and 5 mm/s, respectively.

**Figure 1:**
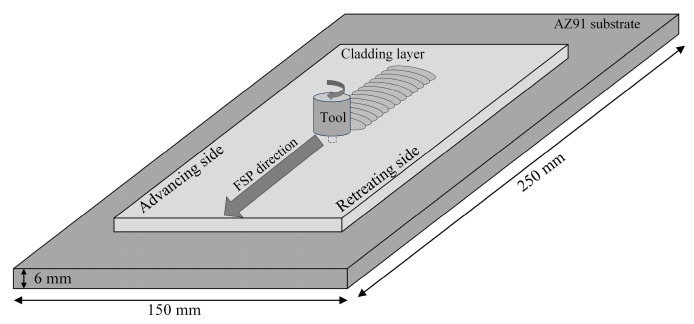
Schematic of the FSP applied to the CMT cladding layer.

The obtained cladding layers were subjected to FSP using a conical threaded stirring head with a shoulder diameter of 15 mm and a pin length of 5 mm. The root diameter and tip diameter of the pin were 6 mm and 4 mm, respectively. As shown in Figure 1, the processing direction of FSP was consistent with the traveling direction of the welding torch during the CMT cladding process. The rotation rate of 300 r/min and the processing speed of 60 mm/min were selected for FSP experiments based on the results of our prior investigation [10].

The cross-sectional metallographic samples of the cladding layer before and after FSP were cut perpendicularly to the processing direction. After grinding and polishing, the metallographic sample was etched with a solution consisting of 10 mL hydrochloric acid and 100 mL alcohol. The microstructure of the cladding layer before and after FSP was observed and analyzed using ZEISS VERT A1 optical microscope (OM, Carl Zeiss AG, Oberkochen, Germany) and JSM-7800F scanning electron microscope (SEM, JEOL Ltd., Tokyo, Japan).

The electrochemical performance of the cladding layer samples before/after FSP was evaluated by the open circuit potential (OCP), potentiodynamic polarization (PDP), and electrochemical impedance spectroscopy (EIS) measurements. The OCP and PDP tests were performed on an electrochemical workstation (ZF-100, Shanghai Zhengfang Electronic Appliances Co., Ltd., Shanghai, China) using a three-electrode cell consisting of a saturated calomel electrode (SCE) as the reference electrode, platinum sheet as the counter electrode, and the specimen as the working electrode in 3.5 wt.% NaCl solution. After grinding with 2000-grit paper, the specimens for electrochemical tests were polished with a metallographic polishing agent with a particle size of 1.5 μm. Then, they were ultrasonically cleaned with deionized water and anhydrous ethanol. The specimens used for the electrochemical corrosion test had an exposed area of 0.25 cm^2^ and the scan rate was set to 0.5 mV/s. EIS measurements were carried out using an electrochemical workstation (PARSTAT 2273, Teledyne Princeton Instruments, Trenton, NJ, USA), and the test frequency ranged from 10 mHz to 100 kHz with a sinusoidal signal amplitude of 5 mV. Unless otherwise stated, all tests were conducted without stirring or deaeration at room temperature of 298 ± 0.2 K.

The dimensions of the sample used in the immersion corrosion test were 5 mm × 6 mm × 10 mm, and the ratio of the surface area (cm^2^) of the sample to the volume of the solution (mL) had to be less than 1:20 during the test [20]. The immersion times were 0.5 h, 1.5 h, 3 h, 6 h, 12 h and 24 h. Each sample under a different immersion time was measured three times, and the average value was taken as the final result. The average corrosion rate *V* (mg·cm^−2^·h^−1^) of the cladding layer before/after FSP under different corrosion periods was calculated by using the weight loss method as follows:

(1) $$V=\frac{m_{0}-{\;m}_{1}}{S_{0}\;\text{×}\;t}$$

where *m*_0_ and *m*_1_ represent the mass of the sample (mg) before and after the immersion test, respectively, *S*_0_ is the surface area of the sample immersed in the solution (cm^2^), and *t* represents immersion time (h).

After the immersion test, the corrosion morphology of the samples before and after removing the corrosion products was observed by SEM, and the element composition of the corrosion products formed on the cladding layer surface was analyzed by energy-dispersive spectrometry (EDS). X-ray diffraction (XRD, D8 Advance, Bruker Corporation, Billerica, MA, USA) tests were conducted in order to identify the phase composition of the corrosion products, and the scanning range was 10° to 90° with a scanning speed of 8°/min.

## 3. Results and Discussion

### 3.1. Electrochemical Measurements

**Figure 2:**
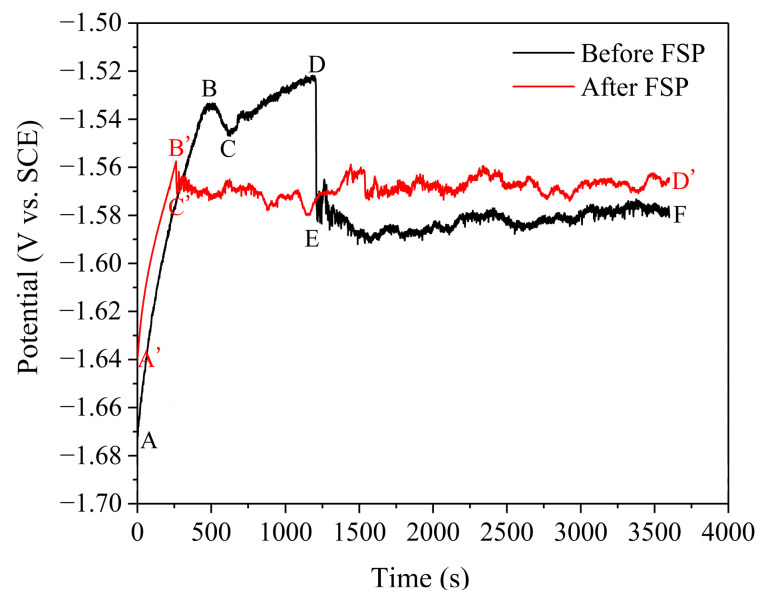
The open circuit potentials of the CMT cladding layer before/after FSP.

Figure 2 shows that the dynamic process of the open circuit potential vs. SCE (E_ocp_) of the AZ91 magnesium alloy cladding layer before/after FSP can be distinguished into two stages, i.e., the initial stage (zone AB and A’B’) and the stable stage (zone EF and C’D’). In the initial stage, the E_ocp_ of both the cladding layer before/after FSP rapidly increased in a short time, indicating the initiation and propagation of the corrosion. The protective film formed by corrosion products on the surface of the cladding layer slowed the speed of the corrosive medium into the substrate, leading to an increase in the E_ocp_ during this stage. However, as shown in zone BC and B’C’ in the figure, the protective film was broken by the continuous erosion of the corrosive medium as the corrosion progressed, resulting in a drop in the E_ocp_ of the cladding layer before/after FSP to varying degrees [21]. It is important to note that the E_ocp_ of the cladding layer before FSP temporarily increased from point C to point D, and then rapidly decreased from point D to point E. This indicated that a certain quantity of protective film was formed quickly on the surface of the cladding layer before FSP after the prior film was broken, thus inducing a temporary protective impact on the substrate. However, the cladding layer before FSP suffered pitting corrosion on the surface due to continuous erosion, leading to a significant decrease in E_ocp_. In the stable stage, the value of E_ocp_ fluctuated within a certain range and reached a dynamic equilibrium, indicating that the protective film on the cladding layer was continuously in the process of cracking and self-repairing [22]. Furthermore, it is evident that the E_ocp_ of the FSPed sample was higher than that of the unprocessed sample, and it can be inferred that FSP can effectively improve the stability and compactness of the protective film on the CMT cladding layer [23], thereby enhancing the corrosion resistance of the cladding layer.

**Figure 3:**
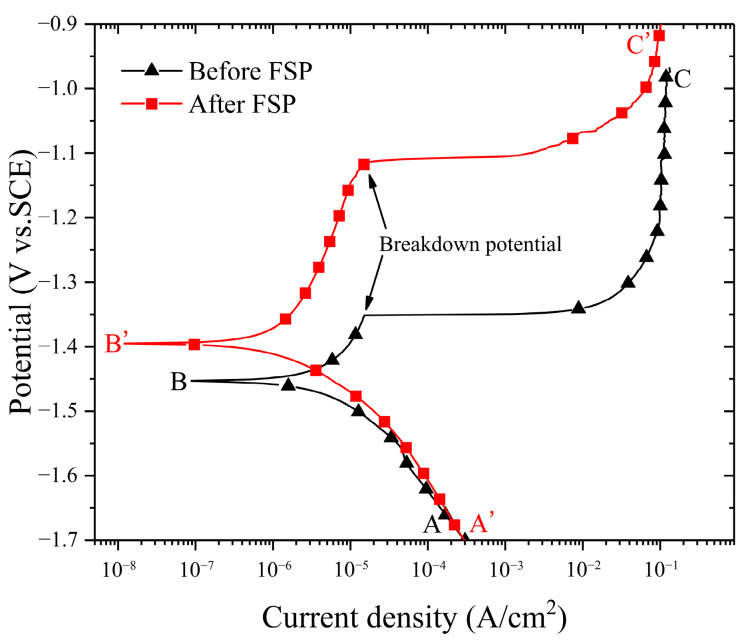
Potentiodynamic polarization curves of the CMT cladding layer before/after FSP.

The potentiodynamic polarization curves and the corresponding Tafel fittings of the CMT cladding layer before/after FSP are depicted in Figure 3. Both curves can be divided into two parts, i.e., cathodic reaction zone and anodic reaction zone. The cathodic reaction, also known as the hydrogen evolution reaction, occurred in the AB and A’B’ zones, while the dissolution of magnesium alloy and an abnormal anodic hydrogen evolution reaction known as the negative difference effect (NDE) [9] occurred in the BC and B’C’ zones (i.e., the anodic zone). The corrosion current density (i_corr_) of the cladding layer was calculated by the Tafel extrapolation method. It was determined that the i_corr_ of the cladding layer after FSP (1.275 μA/cm^2^) was lower than that before FSP (4.135 μA/cm^2^), and this indicated that FSP significantly reduced the corrosion rate of the cladding layer in 3.5 wt.% NaCl solution. As shown in Figure 3, the corrosion potentials (E_corr_) of the cladding layer before and after FSP were −1.455 V and −1.397 V, respectively, indicating that it was harder for the FSPed cladding layer to be corroded compared with the unprocessed cladding layer. Additionally, there were apparent pseudo-passivation stages in the anode branches of the cladding layer when the potential increased beyond point B or point B’. The fact that there was little difference in the i_corr_ of the cladding layer before/after FSP at this stage showed that the corrosion products formed on the layer surface before and after FSP were similar at this time and the corrosion product film was able to prevent further corrosion, leading to a tendency for the i_corr_ to be stable. Compared to the cladding layer before FSP, the pseudo-passivation of the cladding layer after FSP occurred in a larger potential range, and its breakdown potential was also much higher. This indicated that the corrosion product film formed on the cladding layer after FSP had a better protective effect on the substrate of the cladding layer, which was consistent with the results of the OCP measurement. The i_corr_ increased quickly when the potential rose beyond the breakdown potential. When the corrosion product film on the surface of the cladding layer was broken down, the cladding layer continued to dissolve in the corrosive medium rapidly.

In conclusion, FSP enhanced the E_corr_ and breakdown potential and significantly reduced the i_corr_ of the cladding layer, thereby slowing the corrosion rate and improving the corrosion resistance of the cladding layer.

**Figure 4:**
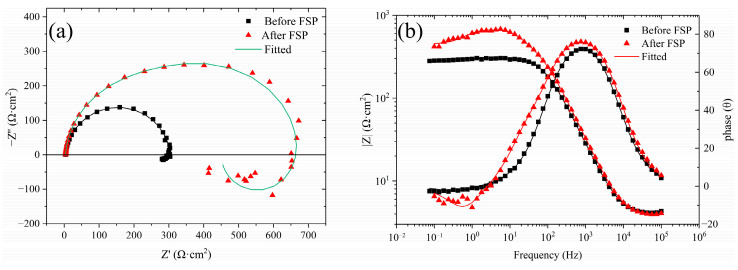
EIS test results of the CMT cladding layer before/after FSP: (**a**) Nyquist plot; (**b**) Bode plot.

The EIS test results for the cladding layers before/after FSP, which were fitted with ZSimWin version 3.3 software, are presented in Figure 4. The Nyquist plot (Figure 4a) shows the inconsistency between the cladding layers before/after FSP, and it is inferred that FSP had an effect on the corrosion behavior of the cladding layer in 3.5 wt.% NaCl solution. There is a large capacitive loop in the high-frequency region and a small inductive loop in the low-frequency region for the cladding layer before FSP. The Bode plot (Figure 4b) shows that the phase of the cladding layer before FSP covered a peak and a less obvious trough, which corresponded to the large capacitive loop and the small inductive loop as above, indicating that there were two time constants for the cladding layer before FSP. As shown in the Nyquist plot of the FSPed cladding layer, there were a large capacitive loop in the high-frequency range, an unapparent small capacitive loop in the intermediate-frequency region, and an inductive loop in the low-frequency range. The corresponding Bode plot shows that the phase of the cladding layer after FSP covered two peaks and an obvious trough, indicating that there were three time constants for the cladding layer after FSP. The large high-frequency capacitive loop represents the charge-transfer process when the cladding layer was corroded, and its diameter indicates the charge-transfer resistance. Generally, the electrode activity correlates negatively with the charge-transfer resistance. The lower the electrode activity of the cladding layer, the less the cladding layer dissolves in the test solution. As shown in Figure 4, the FSPed cladding layer had a greater charge-transfer resistance and a higher impedance modulus over practically the entire frequency range, indicating that FSP dramatically slowed the dissolution rate of the cladding layer in the solution. In addition, the formation of the small medium-frequency capacitive loop of the FSPed cladding layer was induced by the increase in the thickness of the corrosion product on the cladding layer surface [24]. However, the small capacitive loop in the medium-frequency region was not obvious enough due to the loose porous structure of the corrosion product film [9]. However, the emergence of inductive loops in the low-frequency range is often subject to discussions or controversies. The consensus view of most researchers is that inductive loops in the low-frequency range are observed when the electrochemical reactions are coupled with the formation of intermediate species absorbed on the electrode surface. Therefore, it is considered that the intermediate products, such as Mg^+^ and Mg(OH)^+^, adsorbed on the surface of the cladding layer before/after FSP during the anodic reaction were primarily responsible for the inductive loop in the low-frequency region [14].

**Figure 5:**
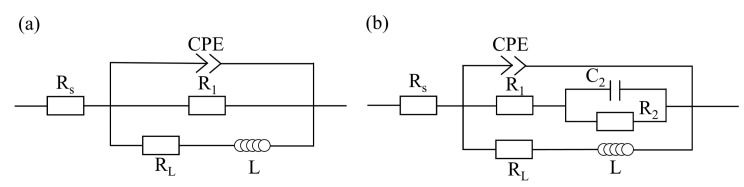
Equivalent circuits for the cladding layer: (**a**) before FSP; (**b**) after FSP.

Figure 5 shows the equivalent circuits for the cladding layer before/after FSP fitted by the EIS data, and the fitting values of the elements in the equivalent circuits are listed in Table 2. The electric double layer (interface between the electrode and electrolyte) is represented by the constant phase angle element (CPE), which is defined by two values of Y and n (dispersion coefficient). If n = 1, the CPE is identical to a capacitor, and if n = 0, CPE represents a resistance. In the fitted circuits, R_s_, R_1_, and R_2_ are solution resistance, the charge-transfer resistance of the cladding layer, and the resistance of the corrosion product film on the cladding layer surface, respectively. L and R_L_ represent the inductance and the inductive resistance, respectively, both of which are used to describe the inductive loop in the low-frequency range. The parallel connection between R_1_ and CPE represents the electric double-layer structure at the interface between the corrosive media and the cladding layer. As shown in Table 2, the R_1_ values of the cladding layer before and after FSP were 300 Ω·cm^2^ and 479.4 Ω·cm^2^, respectively, indicating that the dissolution rate of the cladding layer in the solution decreased after FSP. The decrease in Y value and increase in n value of the CPE can reflect that the corrosion product film structure of the FSPed cladding layer was denser and more uniform [25,26].

### 3.2. Immersion Corrosion Behavior

**Figure 6:**
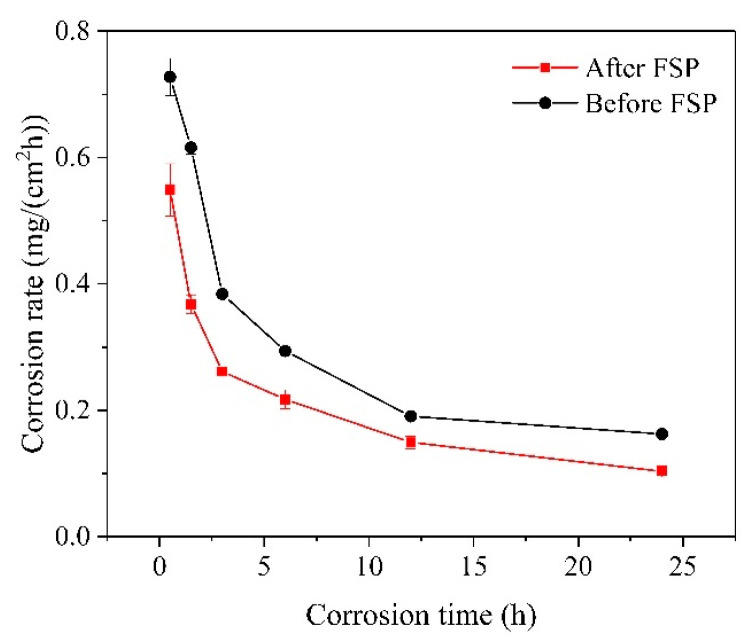
Corrosion rate of the cladding layer before/after FSP in the 3.5 wt.% NaCl solution.

As shown in Figure 6, the corrosion rate curves of the cladding layer before and after FSP had comparable patterns of variation. During the initial stage of the immersion, the corrosion rate was high for both cases and then reduced rapidly after 3 h immersion. After that, the corrosion rate declined gradually and then showed a tendency to be stable. As the immersion time was extended, the corrosion products attached to the cladding layer surface gradually increased, thereby slowing the erosion of the corrosion medium to the cladding layer. In addition, the dissolution of magnesium alloy during the immersion corrosion process led to an increase in the pH value of the solution, resulting in a decrease in the corrosion rate of the cladding layer with the increase in immersion time [27,28].

Moreover, it can be found that the corrosion rate of the FSPed cladding layer was significantly lower than that of the unprocessed sample, which was consistent with the electrochemical measurements results in the previous section. Our previous research indicated that the grains in the FSPed sample were significantly refined, and the elongated β-Mg_17_Al_12_ second phase originally distributing at the grain boundary was fully crushed and dispersed in the α-Mg matrix [10]. The corrosion potential of the β phase with a long strip distribution in the unprocessed sample was higher than that of the α-Mg matrix, so the β phase acted as a cathode during the corrosion process, accelerating the corrosion of the cladding layer substrate. The quantity reduction and dispersed distribution of β phases lessened the local micro-galvanic corrosion, thus greatly slowing down the corrosion rate of the FSPed cladding layer. In addition, the grain refinement induced by FSP led to an increase in the volume fraction of the grain boundaries in the cladding layer, improving the adhesion between the surface of the cladding layer and the protective corrosion product film and ultimately enhancing the corrosion resistance of the cladding layer [29].

**Figure 7:**
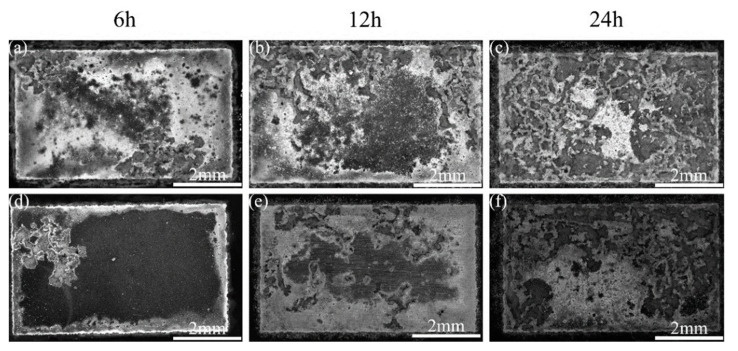
Macroscopic corrosion morphology of the cladding layer after different immersion times: (**a**–**c**) before FSP; (**d**–**f**) after FSP.

Figure 7 shows the macroscopic corrosion morphology of the unprocessed and FSPed cladding layer after immersion for 6 h, 12 h, and 24 h, respectively. After immersion for 6 h, the surface of the cladding layer before FSP had undergone quite severe localized corrosion, and the majority of the surface was covered by corrosion products. For the FSPed cladding layer under the same immersion time, the corrosion region was smaller and its surface was relatively flat, indicating that the degree of corrosion was substantially lower than the former. The deterioration of both types of cladding layers became worse with immersion time. After immersion for 24 h, the surface of the cladding layer before FSP was almost entirely corroded, while there were still some regions on the FSPed cladding layer surface where the corrosion was not noticeable.

To sum up, the corrosion phenomenon occurred when both of the cladding layers before/after FSP were immersed in NaCl solution. However, the corrosion degree of the FSPed cladding layer was significantly weaker compared with the unprocessed cladding layer, indicating that FSP can effectively inhibit the corrosion propagation rate of the cladding layer in NaCl solution, thereby improving its corrosion resistance.

**Figure 8:**
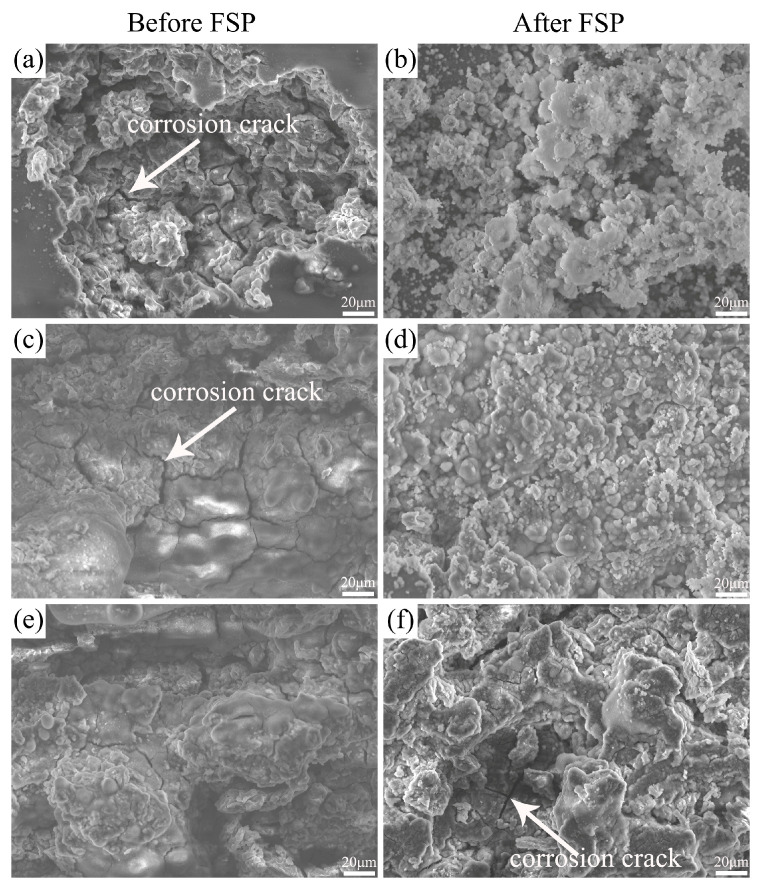
SEM corrosion morphology of the cladding layer before/after FSP after different immersion times: (**a**,**b**) 6 h; (**c**,**d**) 12 h; (**e**,**f**) 24 h.

Figure 8 shows the SEM corrosion morphology of the unprocessed and FSPed cladding layer without removal of the corrosion products after different immersion times. After immersion for 6 h, the surface of the cladding layer before FSP exhibited significant pitting corrosion, accompanied by the formation of corrosion cracks, and spherical corrosion products were formed in some regions on the FSPed cladding layer surface. When the immersion time reached 12 h, the corrosion cracks on the surface of the cladding layer before FSP propagated and connected to each other, leading to partial detachment of the substrate. In comparison, a relatively dense protective film of corrosion products was formed on the surface of the FSPed cladding layer, and no obvious corrosion cracks were observed. After further prolonging the immersion time to 24 h, the surface of the cladding layer before FSP showed pronounced grooves, indicating an extensive detachment of the substrate, and corrosion cracks were observed on the surface of the FSPed cladding layer. As previously mentioned, during the corrosion process, the β phase acted as a cathode due to its higher corrosion potential, accelerating the anodic dissolution of the α-Mg matrix. However, FSP crushed the elongated β-phase in the cladding layer and made it disperse into the α-Mg matrix. As a result, the aforementioned accelerated corrosion effect was significantly weakened.

**Figure 9:**
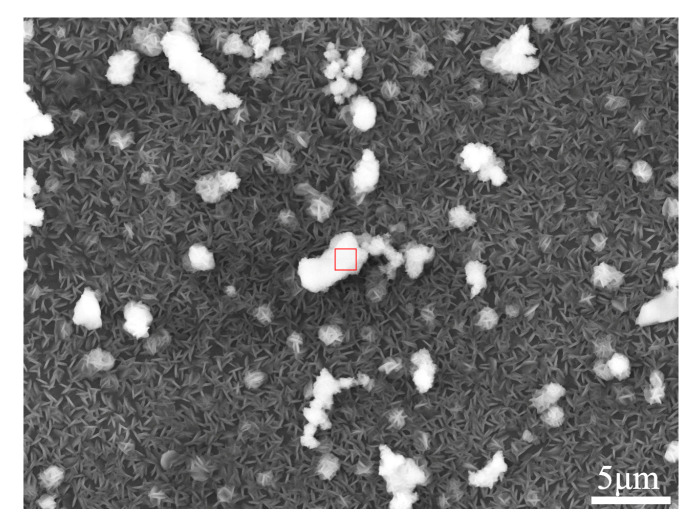
SEM of the corrosion products on the FSPed cladding layer surface.

As shown in Figure 9 and Table 3, the corrosion products formed on the FSPed cladding layer surface consisted mainly of O, Al, and Mg elements. In addition, a small amount of Cl element was observed. This is due to the fact that the NaCl solution served as the corrosive medium and a small amount of MgCl_2_ was formed during the corrosion process [30]. Combined with the relevant research results [31,32], it can be preliminarily determined that the corrosion products formed on the surface of the cladding layer were mainly composed of Mg(OH)_2_.

**Figure 10:**
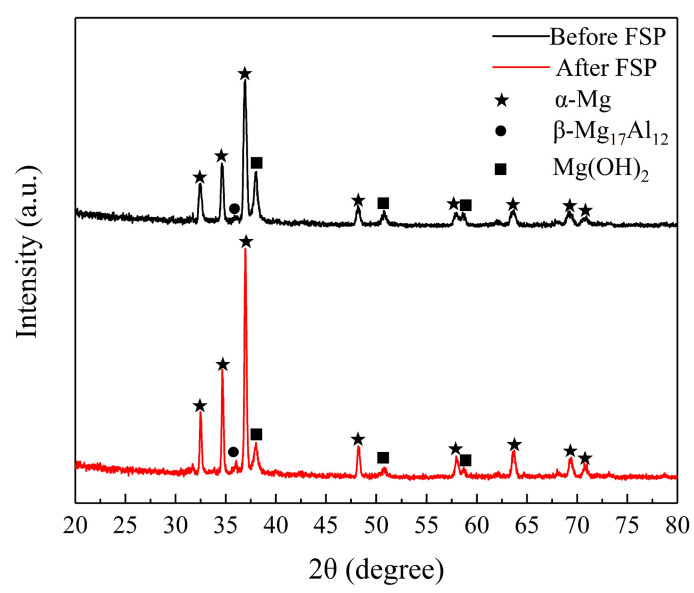
XRD patterns of the corrosion product on the surface of the cladding layer before/after FSP.

XRD analysis was performed to further determine the phase composition of corrosion products formed on the unprocessed and FSPed cladding layer after immersion for 24 h. Figure 10 shows the diffraction peaks of Mg(OH)_2_ that were observed in addition to the α-Mg and β-Mg_17_Al_12_ diffraction peaks, indicating that the corrosion products formed on the surface of the cladding layer were mainly composed of Mg(OH)_2_.

**Figure 11:**
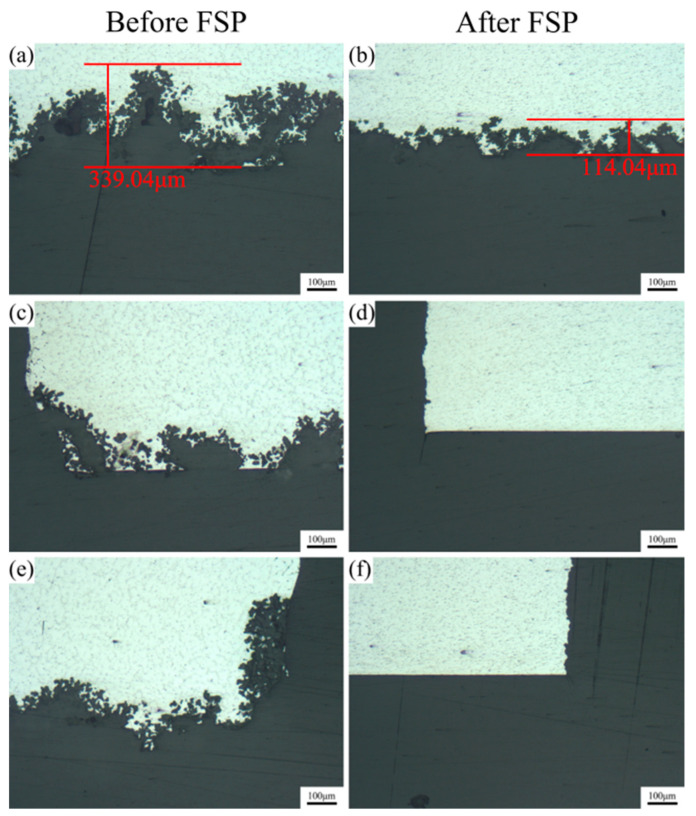
Macroscopic cross-sectional morphology of the corrosion product film on the cladding layer after immersion for 24 h: (**a**,**c**,**e**) before FSP; (**b**,**d**,**f**) after FSP.

Figure 11 shows the cross-sectional morphology of the corrosion product film formed on the unprocessed and FSPed cladding layers after immersion for 24 h. Figure 11a,b shows that the cladding layer before FSP experienced severe localized corrosion with a maximum corrosion depth of 339.04 μm, while the FSPed cladding layer showed a relatively uniform cross-sectional corrosion morphology with a maximum corrosion depth of 114.04 μm. As shown in Figure 11c–f, the two ends at the cross-section of the cladding layer before FSP were severely corroded, while those of the FSPed cladding layer remained relatively intact, without apparent corrosion pits on the surface.

**Figure 12:**
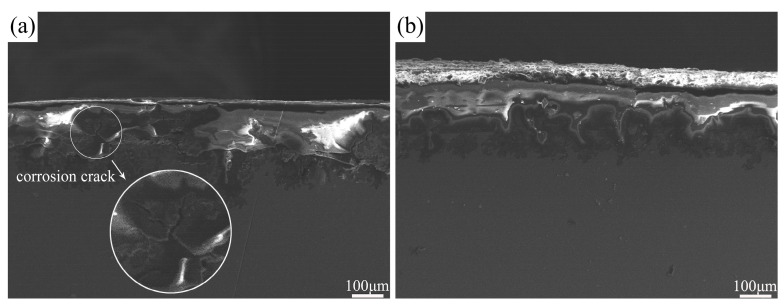
SEM cross-sectional corrosion morphology of the cladding layer after immersion for 24 h: (**a**) before FSP; (**b**) after FSP.

Figure 12 shows the SEM cross-sectional corrosion morphology of the unprocessed and FSPed cladding layer after immersion for 24 h. It can be observed that the cladding layer before FSP exhibited severe corrosion with some large corrosion pits and accompanied by the formation of corrosion cracks. In contrast, the FSPed cladding layer demonstrated significant reduction in corrosion damage along the vertical direction, with relatively uniform corrosion morphology and shallow corrosion pits.

The difference in the cross-sectional corrosion morphology was closely related to the morphological change of the β-Mg_17_Al_12_ second phase in the cladding layer. In the cladding layer before FSP, the long-stripe β-Mg_17_Al_12_ phase distributed at the grain boundaries, with significant gaps. This configuration allowed the formation of corrosion cells between the β-Mg_17_Al_12_ phase and the α-Mg matrix, and the α-Mg matrix acted as the anode and underwent dissolution. Additionally, the large gaps between the second phases hindered their ability to impede the corrosion propagation, resulting in severe corrosion of the cladding layer before FSP. For the FSPed cladding layer, the β-Mg_17_Al_12_ second phase was sufficiently crushed and dispersed into the α-Mg matrix with a granular form, resulting in a significant decrease in the area of the second phase serving as a cathode. Moreover, the distance between the second phases was also significantly reduced, playing an effective role in corrosion blocking [18] and leading to a relatively short corrosion propagation path in the FSPed cladding layer.

In summary, FSP reduced the area ratio of the β-Mg_17_Al_12_ phase as cathode to the α-Mg matrix as anode due to the grain refinement and the changing in the morphological distribution of the β-Mg_17_Al_12_ second phase [33], thereby enhancing the corrosion resistance of the cladding layer. Additionally, the significant reduction in the distance between the β-Mg_17_Al_12_ second phases effectively inhibited the corrosion propagation within the cladding layer matrix, leading to a significant decrease in the dissolution rate of the matrix in NaCl solution. Therefore, the FSP not only improved the mechanical properties of the AZ91 magnesium alloy CMT cladding layer [34] but also enhanced its corrosion resistance in the environment containing Cl^−^.

### 3.3. Corrosion Mechanism

The hydrogen evolution reaction and anodic reaction of the AZ91 magnesium alloy CMT cladding layer in NaCl solution can be expressed as follows:

(2) $${2H}_{2}{O+2e}^{-}\;\to {\;2\mathrm{OH}}^{-}{+H}_{2}$$

(3) $$\mathrm{Mg}\;\to {\;\mathrm{Mg}}^{2+}{+2e}^{-}$$

Then, Mg^2+^ combines with OH^−^ in the solution to form Mg(OH)_2_, so the total reaction formula for the entire corrosion process is:

(4) $${\mathrm{Mg}+2H}_{2}O\;\to {\;\mathrm{Mg}(\mathrm{OH})}_{2}{+H}_{2}$$

However, the corrosion product Mg(OH)_2_ is not stable in aqueous solutions, especially in Cl^−^-containing environments. Cl^−^ can degrade the precipitate of Mg(OH)_2_ by producing MgCl_2_ [4,16]. Therefore, the EDS test results of the corrosion products formed on the cladding layer surface, as shown in Table 3, indicated that the protective corrosion product film on the surface of the cladding layer was broken, resulting in further corrosion of the cladding layer.

**Figure 13:**
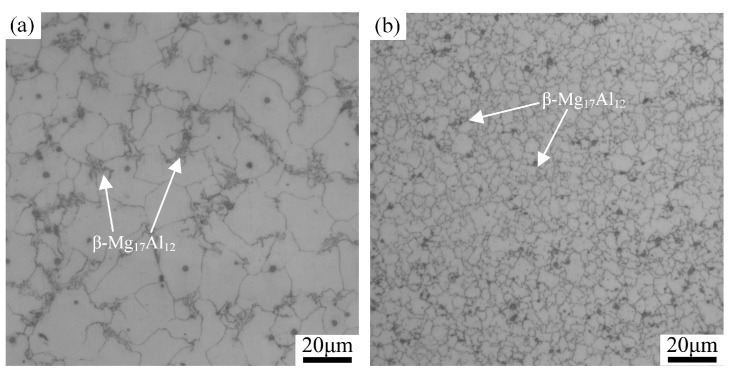
Microstructure of the cladding layer: (**a**) before FSP; (**b**) after FSP.

For the magnesium alloy cladding layer, the α-Mg matrix, serving as the anode, dissolved when the corrosion occurred, and the degree of corrosion damage gradually increased with the extension of time. Al is one of the main components of the AZ91 magnesium alloy, and its content has a certain influence on the corrosion resistance of the cladding layer. Song et al. [27] reported that the corrosion resistance of magnesium alloys increased with an increase in the Al content, because Al effectively reduced the surface reactivity of magnesium alloys. Figure 13 shows the microstructure of the cladding layer before/after FSP, and it can be observed that the FSP caused the fragmentation and dispersion of the β-Mg_17_Al_12_ second phase, which is rich in Al, in the cladding layer. As a result, the content of Al in the α-Mg matrix increased, effectively suppressing the hydrogen evolution and self-corrosion. Therefore, uniform corrosion occurred on the FSPed cladding layer surface with a low degree of corrosion.

In addition, FSP induced severe plastic deformation in the stir zone, leading to dynamic recrystallization of the material. Consequently, the grains were significantly refined, which is considered a key factor in enhancing the corrosion resistance of AZ91 magnesium alloy cladding layers.

The Pilling–Bedworth ratio (P-B ratio) of metal represents the ratio of the volume per metal ion in the oxide film formed on the metal surface to the volume per metal atom in the metal, and it can reflect the stress state of the oxide film on the metal surface. Generally speaking, when the P-B ratio is lower than 1 or higher than 2, excessive tensile stress or excessive compressive stress is generated in the oxide film such that the oxide film is prone to cracking or flaking. When the P-B ratio is in the range of 1 to 2, it means that a stable and protective passivation film can be formed on the metal surface. Due to the P-B ratio of magnesium alloy being less than 1, the corrosion product film formed on its surface is prone to cracking under excessive tensile stress. Therefore, the surface film on the magnesium alloy cladding layer could not provide effective protection for the substrate, leading to further corrosion of the cladding layer in the NaCl solution. FSP significantly increased the grain boundary density of the cladding layer owing to grain refinement such that the mismatch between the substrate and the oxide layer was effectively compensated, leading to an increase in the adhesion between the corrosion product film and the cladding layer substrate and finally significantly improving the corrosion resistance of the cladding layer [35,36].

**Figure 14:**
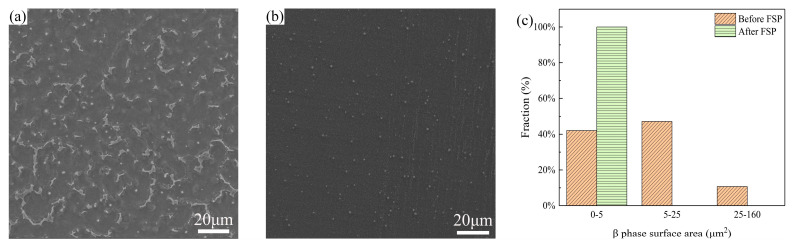
(**a**) SEM morphology of the cladding layer before FSP; (**b**) SEM morphology of the FSPed cladding layer; (**c**) area distribution of β phase in (**b**,**c**).

Figure 14a,b shows the SEM morphology of the cladding layer before/after FSP. In order to explore the size of the change in the β phase, the area of the β phase in both figures was calculated using ImageJ. As shown in Figure 14c, 47.2% and 10.7% of the β phase area in the cladding layer before FSP was 5–25 μm^2^ and 25–160 μm^2^, while the area of all the β phase in the FSPed cladding layer was less than 5 μm^2^.

Due to the potential difference between the β-Mg_17_Al_12_ phase and the α-Mg matrix, the β phase with a higher corrosion potential acts as a cathode to accelerate corrosion. When the α-Mg matrix as the anode was eroded and dissolved by the corrosive medium, the β phase as the cathode also acted as a barrier to corrosion propagation, effectively hindering further corrosion [37]. Therefore, the β phase has a dual role of accelerating and inhibiting corrosion of the magnesium alloy cladding layer, but whether the β phase can act as a corrosion barrier depends mainly on its quantity and distribution. Song et al. [38] pointed out that the β phase with high volume fraction and distribution at the grain boundary in a network shape can act as a barrier layer, thereby slowing the corrosion rate of the magnesium alloy, while the β phase with small volume fraction and dispersed distribution at the grain boundary mainly acts as a cathode, accelerating the dissolution rate of the α-Mg matrix and thus resulting in a decrease in the corrosion resistance of the magnesium alloy. The interval between the adjacent β phases in the cladding layer before FSP was large, so the β phase could not effectively hinder the corrosion propagation in the cladding layer. Although the β phase in the FSPed cladding layer did not continuously distribute at the grain boundary, the distance between the adjacent β phases was significantly reduced due to their dispersed distribution in the matrix, so the erosion of the corrosive medium on the matrix was effectively suppressed.

**Figure 15:**
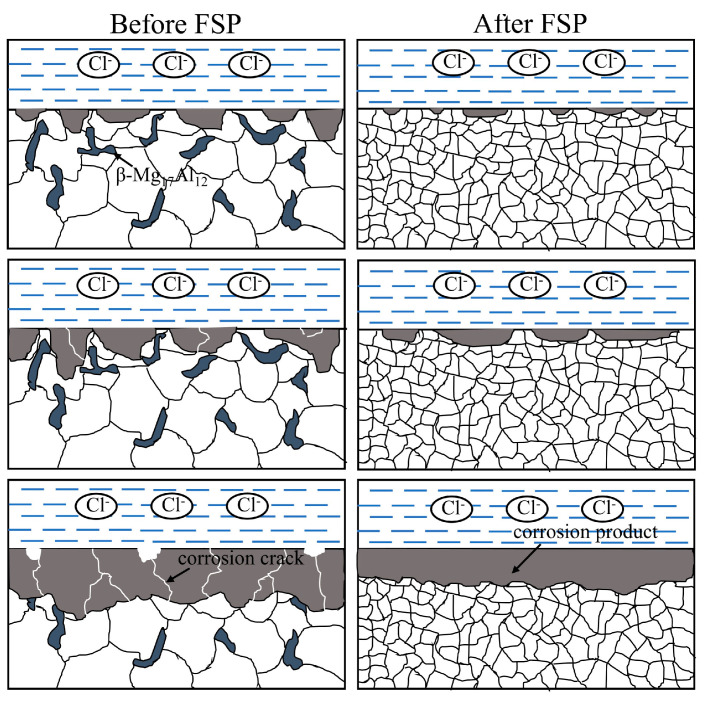
Schematic diagram of the corrosion process of the cladding layer in 3.5 wt.% NaCl solution.

Figure 15 depicts the schematic of the corrosion process of the AZ91 magnesium alloy CMT cladding layer in 3.5 wt.% NaCl solution. The β-Mg_17_Al_12_ phase with long strip shape distributed at the grain boundaries in the cladding layer before FSP. The distance between the β phases was large, and it acted as a cathode when corrosion occurred, thus accelerating the corrosion of the cladding layer. Meanwhile, the coarse grains of the cladding layer before FSP caused poor bonding between the corrosion products film and the substrate, leading to the formation of corrosion cracks. Therefore, severe localized corrosion occurred in the regions around the β phase of the cladding layer before FSP. With the extension of the erosion time, corrosion pits continued to propagate inward through the gap between the β phases. Cracks initiated in the corrosion product film, causing some corrosion products to flake, so the degree of corrosion damage to the substrate of the cladding layer was intensified. Due to the significant grain refinement induced by FSP, the corrosion product film and the substrate of the cladding layer were closely bonded, thereby inhibiting the formation of cracks in the corrosion product film and effectively hindering the Cl^−^ erosion of the cladding layer substrate. Therefore, in the early stage of corrosion, the number of shallow corrosion pits on the surface of the cladding layer was small. Since the β phase was crushed and dispersed into the α-Mg matrix, the area ratio of the β phase serving as the cathode to the α-Mg matrix serving as the anode was significantly reduced, resulting in a decrease in the corrosion rate. In addition, due to the uniform distribution of the β phase in the α-Mg matrix, the distance between the adjacent β phases was significantly reduced, providing a continuous corrosion barrier. Therefore, it was difficult for the corrosion to continue to propagate along the gap between the β phases, thus improving the corrosion resistance of the cladding layer.

## 4. Conclusions

The electrochemical performance and immersion corrosion behavior of the AZ91 magnesium alloy CMT cladding layer before/after FSP in 3.5 wt.% NaCl solution were investigated, and the influence mechanism of FSP on the corrosion resistance of the cladding layer was comprehensively discussed. The main conclusions obtained in this study are as follows.

(1) Compared with the cladding layer before FSP, the corrosion potential and corrosion current density of the FSPed sample increased from −1.455 V to −1.397 V and decreased from 4.135 μA/cm^2^ to 1.275 μA/cm^2^, respectively. The protective corrosion product film formed on the FSPed cladding layer surface was denser, and the breakdown potential of the protective film was significantly increased.
(2) The FSPed cladding layer had a higher charge-transfer resistance, indicating that its dissolution rate in NaCl solution was relatively low. The decrease in Y value and increase in n value of the CPE reflects that the corrosion product film structure of the FSPed cladding layer was denser and more uniform.
(3) The corrosion rate of both the unprocessed and FSPed cladding layers in NaCl solution decreased with the increase in immersion time, and the corrosion rate of the FSPed sample was lower. Severe localized corrosion occurred on the unprocessed cladding layer surface accompanied by the formation of corrosion cracks, while relatively uniform corrosion with shallow corrosion pits occurred on the FSPed cladding layer surface.
(4) An increase in the grain boundary density due to the grain refinement induced by FSP improved the adhesion between the protective film and the cladding layer substrate and facilitated the formation of a denser and more uniform corrosion product film on the cladding layer surface, leading to a decrease in the dissolution rate of the cladding layer. Additionally, due to the uniform distribution of the β phase within the α-Mg matrix in the FSPed cladding layer, the area ratio of cathode to anode decreased during the corrosion process, resulting in a decrease in galvanic corrosion rate.
(5) FSP effectively improved the corrosion resistance of the AZ91 magnesium alloy CMT cladding layer by increasing the Al content in the α-Mg matrix, refining the grains and causing the fragmentation and dispersion of the β phase.

## Figures and Tables

**Table 1:** Chemical compositions of the substrate and deposited wire (wt.%).

Material	Al	Zn	Mn	Si	Cu	Fe	Ni	Mg
Substrate (AZ91)	8.70	0.58	0.24	0.020	0.0050	0.0020	0.00100	Bal.
Wire (AZ91)	8.99	0.65	0.26	0.037	0.0025	0.0018	0.00043	Bal.

**Table 2:** Fitting values of the elements in the equivalent circuits of Figure 5.

Parameter	Before FSP	After FSP
R_s_ (Ω·cm^2^)	4.03	3.86
R_1_ (Ω·cm^2^)	300	479.4
Y (s^n^·Ω^−1^·cm^−2^)	9.22 × 10^−6^	7.22 × 10^−6^
n	0.95	0.96
C_2_ (F·cm^−2^)	/	3.32 × 10^−5^
R_2_ (Ω·cm^2^)	/	198.2
R_L_ (Ω·cm^2^)	2990	1311
L (H·cm^−2^)	1452	415.8

**Table 3:** EDS results of the corrosion product marked in Figure 9.

Element	O	Mg	Al	Cl
wt.%	44.57	41.63	3.16	10.63
at.%	56.68	34.84	2.38	6.10

## Data Availability

Data are contained within the article.
